# Minimal requirements for ISO15189 validation and accreditation of three next generation sequencing procedures for SARS-CoV-2 surveillance in clinical setting

**DOI:** 10.1038/s41598-023-34088-w

**Published:** 2023-04-28

**Authors:** Céline Maschietto, Gaëtan Otto, Pauline Rouzé, Nicolas Debortoli, Benoît Bihin, Lesly Nyinkeu, Olivier Denis, Te-Din Huang, François Mullier, Pierre Bogaerts, Jonathan Degosserie

**Affiliations:** 1 Department of Laboratory Medicine, UCLouvain, CHU UCL Namur, 5530 Yvoir, Belgium; 2 COVID-19 Federal Testing Platform Bis, CHU UCL Namur & UNamur, 5530 Yvoir, Belgium; 3 Laboratory of Microbiology, CHU UCL Namur, 5530 Yvoir, Belgium; 4 Namur Molecular Tech, CHU UCL Namur, 5530 Yvoir, Belgium; 5 Scientific Support Unit, CHU UCL Namur, 5530 Yvoir, Belgium

**Keywords:** Molecular medicine, Next-generation sequencing, SARS-CoV-2

## Abstract

Rapid and recurrent breakthroughs of new SARS-CoV-2 strains (variants) have prompted public health authorities worldwide to set up surveillance networks to monitor the circulation of variants of concern. The use of next-generation sequencing technologies has raised the need for quality control assessment as required in clinical laboratories. The present study is the first to propose a validation guide for SARS-CoV-2 typing using three different NGS methods fulfilling ISO15189 standards. These include the assessment of the risk, specificity, accuracy, reproducibility, and repeatability of the methods. Among the three methods used, two are amplicon-based involving reverse transcription polymerase chain reaction (Artic v3 and Midnight v1) on Oxford Nanopore Technologies while the third one is amplicon-based using reverse complement polymerase chain reaction (Nimagen) on Illumina technology. We found that all methods met the quality requirement (e.g., 100% concordant typing results for accuracy, reproducibility, and repeatability) for SARS-CoV-2 typing in clinical setting. Additionally, the typing results emerging from each of the three sequencing methods were compared using three widely known nomenclatures (WHO, Pangolineage, and Nextclade). They were also compared regarding single nucleotide variations. The outcomes showed that Artic v3 and Nimagen should be privileged for outbreak investigation as they provide higher quality results for samples that do not meet inclusion criteria for analysis in a clinical setting. This study is a first step towards validation of laboratory developed NGS tests in the context of the new European regulation for medical devices and *in vitro* diagnostics.

## Introduction

Coronaviruses are enveloped single-stranded RNA viruses widely distributed among mammals and birds that cause a wide range of diseases including respiratory infections. These viruses have great genetic diversity and a high ability to carry out genetic recombination and mutations^1,2^. At the end of 2019, a new member of the coronavirus subfamily, named SARS-CoV-2, was first detected in China (Wuhan) and became responsible for the unprecedented global pandemic that followed. Despite its low mutation frequency compared to other RNA viruses^3^, the high transmission rate of SARS-CoV-2 in humans increases the chances of mutation acquisition and consequent evolution. Therefore, many variants of SARS-CoV-2 have emerged over time, including variants of interest and variants of concern (VOCs). The latter presents higher transmissibility and/or ability to evade immunity than previously circulating strains^3–6^.

Most countries have tried to control new waves of infection by implementing public health measures with the aim of avoiding the flooding of healthcare facilities, thus ensuring access to healthcare for people in need of essential treatment. In this context, it was essential to develop an efficient surveillance network allowing the early detection of new circulating variants and their transmission in the population^7,8^. Whole genome sequencing not only helps scientists study the virus and develop vaccines but is also the key tool for establishing strong surveillance networks.

The entire SARS-CoV-2 genome was first sequenced in early 2020 using virus infected cells and combining several Next Generation Sequencing (NGS) technologies (i.e. Illumina and Oxford Nanopore Technologies)^9^ . Both technologies are still widely used around the world to obtain sequences of circulating strains^5–7,10,11^. In September 2020, the UK national sequencing network, called Coronavirus Disease 2019 (COVID-19) Genomics UK Consortium, identified the first VOC known today as the alpha variant (B.1.1.7). This variant of SARS-CoV-2 is characterized by the HV69-70 deletion and the N501Y amino acid mutation. It was capable of spreading very quickly and exhibited a total of 14 lineage-specific amino acid replacements compared to the first strain identified in Wuhan, including three mutations known to confer epidemiological benefits (i.e. N501Y, 69-70 deletion, P681H)^7,12^. Since then, four main variants of concern have been identified globally: the beta (K417N, E484K), gamma (K417T, V1176F), delta (L19R, L452R, 157-158 deletion, L452R, T478K, D950N), and the latest omicron (R346K, L452X, F486V) variant. Each of these variants has accumulated mutations giving them evolutionary advantages up to the currently most prevalent omicron variants which have acquired more than 60 mutations compared to the Wuhan reference genome^6,13,14^.

Although many countries established different surveillance strategies to monitor VOCs, NGS became the cornerstone strategy widely adopted. NGS technologies were implemented in many laboratories including medical laboratories. While NGS is a qualitative procedure capable of detecting virtually an unlimited number of targets^15,16^, most standards and recommendations for molecular biology analyses in clinical setting are designed for quantitative single-analyte tests^15^. Thus suitable quality control and validation methods are needed to ensure the reliability of the sequencing data generated by NGS. In May 2017, the European Parliament and Council published new regulations about medical devices (Regulation (EU) 2017/745) and *in vitro* diagnostic medical devices (Regulation (EU) 2017/746), also called IVDR, with the goal of improving the quality and safety of clinical diagnostic. These standards greatly increase the number and the diversity of quality requirements for the validation of laboratory developed tests (LDTs). One of the requirements is that all LDTs must be accredited by a national accreditation body and therefore comply with ISO15189 standards^17–19^. The majority of sequencing methods for rapidly evolving and emerging pathogens being LDTs, their validation is a first step towards compliance with ISO15189 standards and subsequent IVDR.

This work aims to complement published data of SARS-CoV-2 sequencing methods, mainly quality criteria^20^ and external quality assessment report^21^, by improving quality control. Indeed, the methods described hereby have been largely used and evaluated^22–24^ for their limitations^25,26^, but lack validation procedures. The present work proposes a validation guide for SARS-CoV-2 typing standard operating procedures (SOP) using NGS and following ISO15189 accreditation standards. In addition, the typing and single nucleotide variations (SNVs) identification abilities of three validated SOPs were compared. To the best of our knowledge, this study is the first to propose a complete validation process for SARS-CoV-2 Whole Genome Sequencing SOPs complying with ISO15189 standards and leading to the accreditation of the methods by a national accreditation body, a first step towards IVDR implementation in clinical setting.

## Methods

### Sampling

Clinical specimens (nasopharyngeal swab in UTM or Zymo medium) were collected from symptomatic or high-risk contact patients for diagnostic purposes only between May 2021 and January 2022, as recommended by national health authorities at the time, and tested positive for SARS-CoV-2 at CHU UCL Namur or at Clinique Saint-Pierre Ottignies using the Allplex®2019-CoV assay (Seegene, Seoul, Korea) or the TaqPath COVID-19 CE-IVD RT-PCR Kit (Thermo Fisher Scientific; MA, USA) that detect SARS-CoV-2 by targeting respectively four genes (N, E and RdRP/S) or three genes (N, S and ORF1). Fifty-five residual samples with a broad range of N-gene qPCR Ct value (from 12.4 to 35.9) were selected for the technical validation of NGS methods.

### Nucleic acid extraction

Total RNA was extracted directly from the clinical samples using two different methods.

For Illumina sequencing, nucleic acids were extracted using the Hamilton Microlab STARlet automated liquid handling system (Accuramed; Halen, Belgium) with the STARMag Viral DNA/RNA 200 C kit (Seegene; Seoul, South Korea) following manufacturer’s instructions. The whole procedure for twenty-four samples took 1h15 (hands-on-time: 15 min).

For Oxford Nanopore Technologies (ONT), nucleic acids were extracted using the Applied Biosystem$^{\textrm{TM}}$ MagMAX$^{\textrm{TM}}$ Viral/Pathogen II Nucleic Acid Isolation kit (Thermo Fisher Scientific; MA, USA) with the KingFisher Flex96 automated system (Thermo Fisher Scientific; MA, USA). Proteinase K and binding beads were added to clinical specimens previously dispensed in a 96 deep-well plate. The plates were introduced in the KingFisher Flex96 and the MVP_2Wash_200_Flex protocol was used, according to manufacturer’s instructions. The whole procedure for twenty-four samples took 45 min (hands-on-time: 20 min).

### Reverse-transcription

Reverse transcription of RNA extracts was performed using the NEBNext®LunaScript®RT SuperMix Kit 5x (New England Biolabs; MA, USA). Following the Illumina Standard Operating Procedure (SOP), 6 $\upmu$L of RNA were mixed with 2.4 $\upmu$L of the enzyme diluted in 3.6 $\upmu$L of nuclease-free water. According to ONT SOP, 8 $\upmu$L of ARN were mixed with 2$\upmu$L of enzyme. Mixes were incubated at 25 $^{\circ }$C for 2 min (poly-dT primers annealing), at 55 $^{\circ }$C during 20 min for the Illumina SOP or 10 min for ONT SOPs (cDNA synthesis), and finally at 95 $^{\circ }$C for 1 min (enzyme inactivation). The whole procedure for twenty-four samples took 20 min for ONT SOPs and 30 min for the Illumina SOP.

### cDNA amplification

#### Illumina

cDNA was amplified using the EasySeq$^{\textrm{TM}}$ SARS-CoV-2 WGS Library Prep Kit (Nimagen B.V.; Nijmegen, Netherlands), hereafter called Nimagen, using Reverse Complement PCR (RC-PCR) technology that produces short amplicons (around 200 bp). This technology allows amplification of cDNA using two types of primers: target-specific primers with a universal tail, and universal primers coupled to Illumina adapter sequences and to index sequences i5 and i7 for sample identification^27^. Two RC-PCRs (A and B) were performed for each sample with two primer pools that cover the whole SARS-CoV-2 genome. cDNA (5 $\upmu$L) of each sample was mixed with 0.2 $\upmu$L of probe panel A or B separately, 0.8 $\upmu$L of probe dilution buffer, and 10 $\upmu$L of 2x Master mix. Samples were incubated at 98 $^{\circ }$C for 2 min for denaturation of double-stranded DNA. Next, functional targeted index primers (combination of target-specific primers and universal primers) were generated by incubation of samples at 98 $^{\circ }$C for 10 s, at 80 $^{\circ }$C for 1 s, at 58 $^{\circ }$C for 10 min and at 72 $^{\circ }$C for 1 min. Then, indexes, sequence adapters and universal tails were added to 5’ and 3’ ends of target DNA fragments by performing two cycles of incubation at 98 $^{\circ }$C for 10 s, 80 $^{\circ }$C for 1 s, 62 $^{\circ }$C for 90 min and for 2 min at 72 $^{\circ }$C. Finally, cDNA fragments with indexes, sequence adapters and universal tails were amplified by performing 40 cycles of incubation at 95 $^{\circ }$C for 10 s, 80 $^{\circ }$C for 1 s, 62 $^{\circ }$C for 2 min and 72 $^{\circ }$C for 1 min. The whole procedure for twenty-four samples took 7 h (hands-on-time: 30 min).

#### ONT

cDNA was amplified with two multiplexed PCRs, each comprising a pool of specific primers (A and B) (Integrated DNA Technologies; Iowa, USA) to cover the whole SARS-CoV-2 genome. Either with short amplicons around 400bp for the Artic nCoV 2019 sequencing SOP v3^22^, hereafter called Artic v3 or with longer amplicons around 1200 bp for the nCoV 2019 rapid barcoding sequencing SOP v1^23^, hereafter called Midnight v1. 2.5 $\upmu$L of cDNA was mixed with 0.4 $\upmu$L (Artic v3 SOP) or 0.11 $\upmu$L (Midnight v1 SOP) of primer pools A or B separately, 12.5 $\upmu$L of Q5®Hot Start High-Fidelity 2X Master Mix (New England Biolabs; MA, USA), and 9.6 $\upmu$L (Artic v3 SOP) or 9.89 $\upmu$L (Midnight v1 SOP) of nuclease-free water and incubated at 98 $^{\circ }$C for 30 s (heat activation), followed by 30 (Artic v3 SOP) or 32 cycles (Midnight v1 SOP) of incubation at 98 $^{\circ }$C for 15 s and 65 $^{\circ }$C for 5 min (primers annealing and elongation). The whole procedure for twenty-four samples took 3h15 (hands-on-time: 30 min) for both SOPs.

### Library preparation

#### Illumina with Nimagen amplicons

All RC-PCR amplified cDNA A and B products were pooled separately. To homogenize the read depth between samples during sequencing, the volume of sample introduced in the pools depends on the viral load of each sample: 2 $\upmu$L if qPCR Ct value <20, 4 $\upmu$L if 20$\le$ qPCR Ct value $\le$25 or 8 $\upmu$L if qPCR Ct value > 25. 40 $\upmu$L of each pool diluted with 60 $\upmu$L of RC-PCR Low-TE buffer (Nimagen B.V.; Nijmegen, Netherlands) was purified using 85 $\upmu$L of Solid Phase Reversible Immobilization (SPRI) AmplicleanTM magnetic beads (Nimagen B.V.; Nijmegen, Netherlands) by mixing DNA and beads (beads:DNA ratio = 0.85), followed by incubation of the mix at room temperature (RT) for 5 min, then beads were washed twice with 75% ethanol. Beads covered with DNA fragments of interest were mixed with 110 $\upmu$L of RC-PCR Low TE Buffer (Nimagen B.V.; Netherlands) and incubated at RT for 2 min. The purification procedure was repeated a second time. Eluted DNA from each pool was quantified using the 1xdsDNA HS kit (Thermo Fisher Scientific; MA, USA) with the Qubit 4.0 (Thermo Fisher Scientific; MA, USA) and amplicons size and integrity were checked using the QIAxcel fragment analyzer (Qiagen; Hilden, Germany) with the DNA Screening Kit (Qiagen; Hilden, Germany).

Each DNA suspension was diluted to reach concentration of 2 nM using RC-PCR Low-TE buffer (Nimagen B.V.; Nijmegen, Netherlands) before being pooled together and diluted to reach a final library concentration of 80 pM. The whole procedure took 1h45 (hands-on-time: 1h30).

#### ONT with Artic v3 amplicons

Amplified DNA from each PCR reaction was pooled by sample and each sample was quantified using the 1XdsDNA HS kit (Thermo Fisher Scientific; MA, USA) and the Qubit 4.0 (Thermo Fisher Scientific; MA, USA). Samples were diluted to obtain at most a factor 2 between the least concentrated sample and the most concentrated one in order to homogenize the read depth. 3.3 $\upmu$L of DNA was blunt-ended with 5’-phosphates and 3’-hydroxyls by addition of 1.2 $\upmu$L of Ultra II End prep reaction buffer, 0.5 $\upmu$L of Ultra II End prep enzyme mix from the NEBNext®End Repair kit (New England Biolabs; MA, USA) and 5 $\upmu$L of nuclease free water and incubated at 25 $^{\circ }$C for 15 min, then at 65 $^{\circ }$C for 15 min. 0.75 $\upmu$L of each blunt-ended sample was barcoded with 1.25 $\upmu$L of native barcodes from the Native Barcoding kit Expansion 96 (Oxford Nanopore Technologies; Oxford, UK), 5 $\upmu$L of Blunt/TA Ligase enzyme (New England Biolabs; MA, USA) and 3 $\upmu$L of nuclease free water by incubating the mixes at 25 $^{\circ }$C for 20 min, then at 65 $^{\circ }$C for 10 min. All samples were pooled and then purified with AMPure XP magnetic beads (Beckman Coulter; CA, USA) by mixing the DNA library with beads at 0.4 beads:DNA ratio and incubated at RT for 8 min. Beads were washed twice with short fragment buffer (Oxford Nanopore Technologies; Oxford, UK) and once with 70% ethanol. DNA fragments were eluted by incubating the beads in 30$\upmu$L of nuclease-free water at RT for 5 min.

The DNA library was quantified using the 1xdsDNA HS kit (Thermo Fisher Scientific; MA, USA) with the Qubit 4.0 (Thermo Fisher Scientific; MA, USA), and 30 ng of double-stranded DNA was ligated to adapter protein AMII (Oxford Nanopore Technologies; Oxford, UK) using 10 $\upmu$L of buffer and 5 $\upmu$L of ligase from NEBNext Quick Ligation Module kit (New England Biolabs; MA, USA) and incubation for 10 min at RT. The DNA library was purified a second time with AMPure XP magnetic beads (Beckman Coulter; CA, USA) to eliminate DNA fragments of very high molecular weight and unattached proteins by mixing the library with beads (bead:DNA ratio = 1) and incubation at RT for 5 min. Beads were washed twice with short fragment buffer (Oxford Nanopore Technologies; Oxford, UK) and DNA was eluted by incubating the beads in 15 $\upmu$L of elution buffer (Oxford Nanopore Technologies; Oxford, UK) for 2 min at RT. The final library was quantified using the 1xdsDNA HS kit (Thermo Fisher Scientific; MA, USA) and Qubit 4.0 (Thermo Fisher Scientific; MA, USA). The concentration of double-stranded DNA must be at least 1ng/$\upmu$L to proceed with sequencing. 37.5 $\upmu$L of Sequencing Buffer and 25.5 $\upmu$L of Loading Beads included in the Sequencing Auxiliary Vials kit (Oxford Nanopore Technologies; Oxford, UK) were added to the library according to manufacturer’s instructions. The whole procedure took 3h30 (hands-on-time: 2h30).

#### ONT with Midnight v1 amplicons

Amplified DNA from each PCR reaction (A and B) was pooled by sample. Samples were barcoded using the Rapid Barcoding Kit 96 (Oxford Nanopore Technologies; Oxford, UK) by incubating 5 $\upmu$L of each sample with 2.5 $\upmu$L of a unique barcode and 2.5 $\upmu$L of nuclease free water at 30 $^{\circ }$C for 2 min, then at 80 $^{\circ }$C for 2 min. Identical volumes of each barcoded sample were pooled. The DNA library was purified by SPRI with magnetic beads (Oxford Nanopore Technologies; Oxford, UK) by mixing DNA and beads (bead:DNA ratio = 1) and incubating the mix at RT for 5 min. Beads were washed twice with 80% ethanol and incubated in 30 $\upmu$L of elution buffer (Oxford Nanopore Technologies; Oxford, UK) to elute DNA. Purified DNA was quantified using the 1XdsDNA HS kit (Thermo Fisher Scientific; MA, USA) and the Qubit 4.0 (Thermo Fisher Scientific; MA, USA). Between 600 ng and 800 ng of DNA library were mixed with 1 $\upmu$L of adapter protein RAPF (Oxford Nanopore Technologies; Oxford, UK) and incubated 5 min at RT. 37.5 $\upmu$L of Sequencing Buffer II and 25.5 $\upmu$L of Loading Beads II included in the Sequencing Auxiliary Vials kit (Oxford Nanopore Technologies; Oxford, UK) were added to the library according to manufacturer’s instruction. The whole procedure took 50 min (hands-on-time: 40 min).

### Sequencing

#### Illumina

The library was loaded into a 300-cycle iSeq100 cartridge (Illumina; San Diego, CA, USA) with an ISeq100 sequencing system (Illumina; San Diego, CA, USA). The following parameters were used for sequencing:

GenerateFastq mode

Library Prep Kit: Nimagen IDX96-U01

Read type: paired-end

Read lengths: read1 = 151, index1 = 10, index2 = 10, read2 = 151

Adapter trimming on

#### ONT

The R9.4 flow cell (Oxford Nanopore Technologies; Oxford, UK) was primed using the Flow Cell Priming kit (Oxford Nanopore Technologies; Oxford, UK) and the library loaded onto the flow cell. Sequencing runs were performed with a GridION (Oxford Nanopore Technologies; Oxford, UK) using the following parameters.

Initial bias voltage: −180mV

Basecall model: High accuracy basecalling

Barcoding Artic v3 library: barcoding_kits=EXPNBD196,trim_barcodes=“off”,require_barcodes_both_ends=“on”, detect_mid_strand_barcodes=“off”,min_score=60

Barcoding Midnight v1 library: barcoding_kits=SQK-RBK11096, trim_barcodes=“off”, require_barcodes_both_ends=“off”, detect_mid_strand_barcodes=“on”, min_score=60, min_score_mid=50

Read filtering: min_qscore=9

### Data analyses

#### Illumina data

The analysis of the reads was carried out according to Jordy Coolen’s easyseq_covid19 pipeline available on GitHub (https://github.com/JordyCoolen/easyseq_covid19). After sequencing, the reads obtained were filtered with fastp (version 0.20.1) based on the quality of the sequences. Reads were aligned to the SARS-CoV-2 reference genome NC_045512.2 with bwa software (version 0.7.17). The sequences corresponding to the primers were clipped off with bamclipper (version 1.0.0). The Single Nucleotide Variants (SNVs) were called using bcftools (version 1.9) combined with KMA (version 1.3.9) with the following parameters: mutation frequency $\ge$ 75%, quality score $\ge$ 20, depth of reads $\ge$ 5. Finally, the consensus sequence was obtained with bcftools consensus (version 1.9)^24^.

#### ONT data

Sequencing data analysis was performed following the instructions of Oxford Nanopore Technologies. Basecalling and demultiplexing (distribution of reads) were carried out in real time by, respectively, guppybasecaller and guppybarcoder, both integrated in the MinKnow sequencing software (version 21.05.12).

Further data processing was performed following the bioinformatics pipeline described by ARTICnetwork^28^. The reads were filtered according to their quality (quality score>9) and their length (Artic: minimum length= 400 bp - maximum length= 700 bp, Midnight: minimum length= 600 bp - maximum length= 1,200 bp) to avoid chimeric reads. The passed reads were mapped to the reference genome of SARS-CoV-2 MN908947.3 using minimap2 (version 2.18-r1015) and SNVs were called for positions with depth of reads $\ge$ 20, and consensus sequence obtained using medaka (version 1.4.3, model r941_prom_variant_g360) a software using trained neural network-based models to appropriately call sequence variations^29^.

#### Typing

The molecular typing of SARS-CoV-2 strains was carried out using different classifications: WHO, Nextclade and Pangolin with Nextclade^30^ (version 2.2.0) and Pango-designation^31^ (version 1.9, lineages version 2022-05-13) databases available online.

### Statistical analyses

Cohen’s kappa was used to measure the pairwise concordance between two lists of SNP calls, say A and B, one for each SOP. It was computed as follows.

Each position is classified as mutated or not according to A and B, which gives a list of paired classifications falling into four categories: (a) mutated for A and B, (b) mutated for A but not for B, (c) mutated for B but not for A and (d) not mutated for either A or B.

The proportion of concordant results (P0) and the proportion of concordant results attributable to chance (Pc) where computed from the number of pairs of each category, na, nb, nc and nd.

$$\begin{aligned} \\& \quad {P0 = \frac{(na + nd)}{N} } \\& \quad {Pc = \frac{(na + nb)*(na+nc)+(nc+nd)*(nb+nd)}{N^2}} \end{aligned} $$

Where N is the total number of pairs. Cohen’s Kappa ($\kappa$) is then defined as follows:

$$\begin{aligned} \kappa = \frac{(P0-Pc)}{(1-Pc)} \end{aligned}$$

The result is a number between −1 and 1, a $\kappa$ of 1 represents a perfect match between two lists while 0 or less represents an agreement that does not exceed what chance would produce. [0;0.2[ indicates very weak agreement, [0.2;0.4[ weak agreement, [0.4;0.6[ moderate agreement, [0.6;0.8[ high agreement, [0.8;1[ very high agreement.

These concordance analyses were performed using R 4.1 (R Foundation for Statistical Computing; Austria, Vienna, 2021).

### ISO15189 accreditation

The ISO 15189:2012 standard specifies quality and competence requirements in medical laboratories^32^. It is used by national accreditation bodies to assess, recognize, and confirm the competence of a medical laboratory for a specific method. This standard requires validation of any non-standard analytical method to confirm its performance and specific requirements for its intended use. The performance characteristics that should be assessed in a qualitative method are:

- Uncertainty, by evaluating the influence of external factors on the result. We used the 5M method, also called Ishikawa diagram, to show that external factors don’t influence the outcome of our analyses. This method is frequently used in molecular biology and is the most used method to assess risk factors in pharmaceutical manufacturing^33^. It eases the systematic assessment of input factors potentially affecting method performance^33,34^.
- Specificity, by verifying that NGS reads for SARS-CoV-2 PCR negative samples do not match SARS-CoV-2 genome.
- Accuracy, by participating to External Quality Assessment (EQA) round trials: we must reach 100% concordance for typing results.
- Reproducibility, by comparing the identified nucleotide mutations for at least 3 samples in 3 independent sequencing runs: we must reach 100% concordance for typing results and $\kappa$ > 0.81 for SNV identification.
- Repeatability, by comparing the identified nucleic acid mutations for at least 3 samples sequenced three times within the same sequencing run: we must reach 100% concordance for typing results and $\kappa$ > 0.81 for SNV identification.

The acceptance thresholds for each characteristic are described in our internal procedure for the validation of molecular biology methods accredited by the Belgian Accreditation Body (BELAC).

### Author statement

As per the Belgian law published in the “Moniteur belge” on December 30th 2008 page 68774 regarding the collection and use of human material for human medical applications or scientific research, no patient consent or ethics committee approval is required for the use of residual diagnostic samples for non-medical research (Title II ; Chap. 1 ; Art. 3 ; §5).

## Results

In this study, we validated and compared three amplicon-based SARS-CoV-2 whole genome sequencing SOPs. In the framework of these validations, fifty-five samples tested positive for SARS-CoV-2 have been sequenced with three sequencing SOPs: Artic v3, Midnight v1 and Nimagen. As part of the accreditation to the ISO15189 standards, we evaluated the uncertainty of each method using the 5M diagram, and the specificity by sequencing three samples from nasopharyngeal swabs tested negative for SARS-CoV-2. Two samples were part of an external quality assessment panel and were used to assess the accuracy of the methods. Seven samples were sequenced in three independent sequencing runs and three times in the same sequencing runs for each SOP to assess, respectively, the reproducibility and repeatability of the methods.

Finally, the typing results and SNVs identification were compared between each SOP.

### Uncertainty (including assessment of cross-contamination)

We evaluated the uncertainty of the SOPs using a 5M diagram (Measurement/Medium, Material, Machine, Method, Manpower) (Supplementary table 1).

The environment (Management/Medium) is under monitored temperature conditions. Rooms have dedicated functions and workbenches are cleaned before and after each manipulation with DNA-Erase Decontamination Solution (MP Biomedicals;Irvine, CA) as described in section “Management/medium” of Supplementary table 1 in order to lower the risk of cross-contamination. Nonetheless, the use of amplified material as well as the large size of sample batches increase the risk of cross-contamination^35^. A negative control (nuclease-free water) was added to each sequencing run, from the reverse transcription step, to assess possible cross-contamination. It was shown that below 5% contaminant reads, no variation in typing or SNVs calling was observed^23^. We reproduced these experiments *in silico* and confirmed the conclusions from Freed et al. (data not shown). The number of reads obtained in our negative controls represents less than 2.5% of the total number of reads obtained for the sample with the lowest number of reads in each run (Artic v3: 1.68% ± 0.011, Midnight v1: 1.90% ± 0.0035, Nimagen: 2.04% ± 0.0031). We can conclude that the three SOPs are sufficiently stringent to reduce technical cross-contamination to a level that does not impact the results.

Reagents batches are documented for each run and the material safety data sheets are available for each reagent (Material). The devices go through daily, quarterly and/or yearly maintenance procedures and software are validated after each major update (Machine). Results go through validation criteria described in the material and methods section before being technically and medically validated, and procedures are revised every two years (Method). The staff is trained, qualified, aware of procedures and evaluated on a yearly basis (Manpower) (Supplementary Table 1).

### Specificity

We evaluated the specificity of the three SOPs by including three SARS-CoV-2 PCR negative samples in three independent runs from extraction to sequencing step. Although these samples generated a high number of reads, up to 68,000 reads, less than ten reads per sample match the SARS-CoV-2 genome (data not shown). This indicates that the three SOPs are highly specific for SARS-CoV-2 sequencing and analyses.

### Accuracy

To assess the accuracy of the three SOPs, we used two samples (UZL_CV10_07 and UZL_CV10_89) received in November 2021 in the context of an External Quality Assessment (EQA) panel organized by the Belgian National Reference Center (NRC) for respiratory viruses as a ring test. Samples originally sequenced in independent labs were centralized by the NRC, typing result was confirmed, and samples were dispatched in two other independent labs. Therefore, we compared our results to those obtained by the originating laboratory and another lab for both samples. The comparison was carried out at two levels: typing according to WHO guidelines and two databases (Pangolin^31^ and Nextclade^30^), and comparison of amino acid mutations. The typing results are identical for sample UZL_CV10_07 in our lab and both labs to which it has been compared for the three nomenclatures (Table 1), while mutation I2230T was only identified by one independent laboratory and ours using the three SOPs (data not shown). Typing results for sample UZL_CV10_89 are identical in our lab and both labs to which it has been compared when using Nextclade or according to WHO guidelines but differ from lab to lab by using the Pangolin database (Table 1). These discrepancies are due to the use of a new version of the Pangolin database in our lab. Indeed, the typing results of both external labs were released using the Pangolin v3.1.16, lineages version 2021-11-25 which did not yet contain AY.126 lineage. Although many discrepancies were observed when analyzing the amino acid mutations described by each lab (data not shown), they did not impact the typing results. The three SOPs showed a 100% success rate. Correct identification of all EQA samples at our lab was confirmed by the NRC. Since then, we successfully participated in three other ring tests organized by the NRC.

**Table 1:** Accuracy assessment: comparison of sequencing results in the context of an EQA round trial organized by the NRC for two samples typed by our lab and by two independent labs.

Testing lab		Laboratory 1	Laboratory 2	Laboratory 3	Laboratory 4	CHU UCL Namur	CHU UCL Namur	CHU UCL Namur
Extraction method		N. A.	Seegene- STARMag Universal Cartridge (Allplex SARS-CoV-2 One-Step Protocol)	N. A.	Maxwell RSC Viral-Total Nucleic Acid Purification kit /MaxWell RSC-system (Promega)	MagMAXTM Viral/Pathogen Kit (KingFisherTM)	MagMAXTM Viral/Pathogen Kit (KingFisherTM)	Seegene- STARMag Universal Cartridge (Allplex SARS-CoV-2 One-Step Protocol)
Sequencing Method		ONT	Illumina CleanPlex SARS-CoV-2 Flex Panel-SOPHiADDM	Ampliseq- Illumina	ONT-GridION Artic Network	ONT: SARS-CoV2 genome sequencing protocol v1200 “midnight”	ONT: SARS-CoV2 genome sequencing protocol v3	Illumina: EasySeq SARS-Cov-2 WGS-Nimagen
Sample UZL_ CV10_07	WHO	Alpha	Alpha	–	–	Alpha	Alpha	Alpha
	NextClade	20I	20I	–	–	20I	20I	20I
	Pangolin	B.1.1.7	B.1.1.7	–	–	B.1.1.7	B.1.1.7	B.1.1.7
Sample UZL_ CV10_89	WHO	–	–	Delta	Delta	Delta	Delta	Delta
	NextClade	–	–	21J	21J	21J	21J	21J
	Pangolin	–	–	AY.39	B.1.617.2	AY.126	AY.126	AY.126

Laboratories 1 and 2 sequenced and analyzed sample UZL_CV10_07 and laboratories 3 and 4 sample UZL_CV10_89. Both samples were sequenced and analyzed using the three different methods in our lab.

### Reproducibility

We evaluated the reproducibility of the three SOPs by sequencing seven samples in three independent runs. Reproducibility was assessed at three levels: typing according to WHO guidelines and two databases (Pangolin and Nextclade), amino acid (aa) mutations and SNVs identification.

Concerning the typing of samples with a sufficient coverage, concordance reached 100% for the three nomenclatures with the three SOPs (Supplementary Table 2). Sample CV2031641758, which had a qPCR Ct value = 29.01, could not be typed in any of the databases used for the Midnight SOP due to very low coverage (run 1: mean coverage= 0x, 0% > 100x, run 2: mean coverage= 0x, 0% > 100x, run 3: mean coverage= 5x, 1.5% > 100x).

Concordance at the aa mutation level was assessed using Cohen’s kappa coefficient. This coefficient ($\kappa$) represents the reliability between two qualitative measures by considering the probability that the agreement occurs by chance. Each SOP had a very high reproducibility for the identification of aa mutations for samples with qPCR Ct values <25 (Artic v3: mean $\kappa$ = 0.9883 ± 0.01011, Midnight v1: mean $\kappa$ = 1 ± 0, Nimagen: mean $\kappa$ = 0.8566 ± 0.03534). For sample with qPCR Ct value = 29.01, the reproducibility is high for Nimagen ($\kappa$ = 0.7599 ± 0.1293), moderate for Artic v3 ($\kappa$ = 0.4945 ± 0.1014) and very weak for Midnight v1 ($\kappa$ = 0 ± 0).

**Figure 1:**
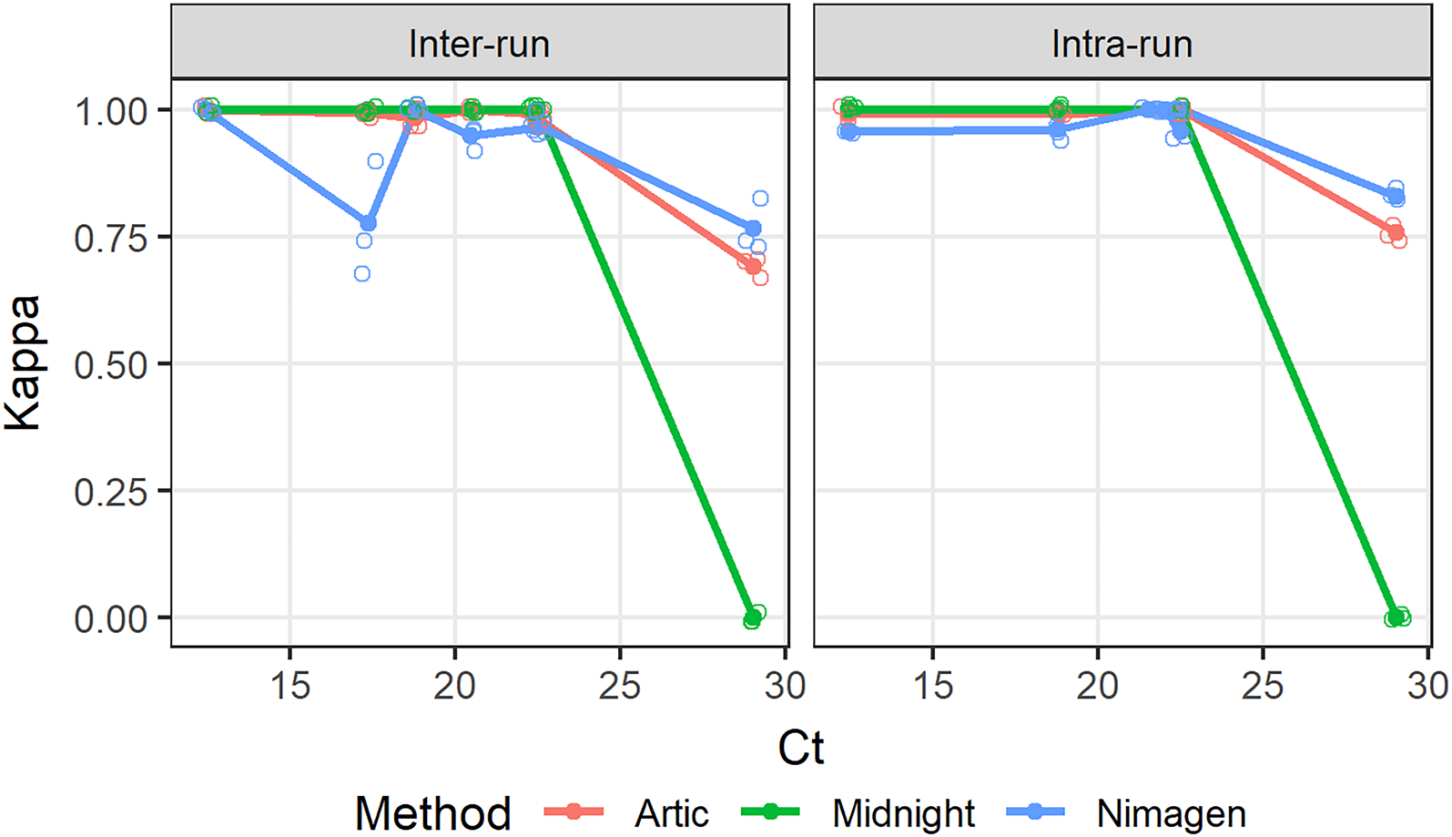
Evolution of Cohen’s kappa coefficient of the reproducibility (left panel) and the repeatability (right panel) for each sample depending on the Ct value for the N-gene and for each method: Artic v3 (red), Midnight v1 (green) and Nimagen (blue). A kappa coefficient of 1 means a perfect match between three independent runs (left panel) or between three repeats in the same run (right panel) when a kappa coefficient of 0 can be interpreted as a agreement that does not exceed what chance would produce. The three methods have high kappa coefficient for reproducibility and repeatability for all samples with Ct value <25 but this coefficient decreases for samples above that threshold.

At the nucleotide mutation level, the reproducibility is high ($\kappa$ > 0.8) for all samples with a qPCR Ct value < 25 (Artic v3: mean $\kappa$ = 0.9918 ± 0.01060, Midnight v1: mean $\kappa$ = 1 ± 0, Nimagen: mean $\kappa$ = 0.9433 ± 0.1468). For the sample with the highest qPCR Ct value (Ct = 29.01), coefficients are lower (Artic v3: $\kappa$ = 0.6896 ± 0.02007, Midnight v1: $\kappa$ = 0 ± 0.00005, Nimagen: $\kappa$ = 0.7659 ± 0.05119) indicating a greater discrepancy, especially for the Midnight v1 SOP. Both short-amplicon methods (Artic v3 and Nimagen) have a coefficient indicating a high correlation (Fig. 1 left panel). Although the reproducibility of the three methods is similar for samples with a qPCR Ct value <25, the reproducibility of the Midnight v1 SOP is significantly different from that of both other methods for samples with a qPCR Ct value >25 (Supplementary table 3).

### Repeatability

We evaluated the repeatability of each SOP by sequencing seven samples three times in a same run. The repeatability was assessed at three levels: typing according to WHO guidelines and two databases (PangoLineage and Nextclade), aa mutations and SNVs identification.

All typing results were concordant for each sample processed with the three SOPs, except for sample CV2031641758 that has the higher qPCR Ct value (29.01) and could not be typed using the Midnight SOP due to low coverage (repeat 1: mean coverage= 1x, 0% > 100x, repeat 2: mean coverage= 0x, 0% > 100x, repeat 3: mean coverage= 1x, 0% > 100x) (Supplementary table 4).

The kappa statistical analysis carried out to evaluate the concordance for aa mutations showed the very high repeatability of all SOPs for samples with qPCR Ct values < 25 (Artic v3: mean $\kappa$ = 0.9953 ± 0.00402, Midnight v1: mean $\kappa$ = 1 ± 0, Nimagen: mean $\kappa$ = 0.9435 ± 0.02546) but the $\kappa$ coefficient decreased for sample with qPCR Ct value > 25 (Artic v3: $\kappa$ = 0.7279 ± 0.08269, Midnight v1: $\kappa$ = 0 ± 0, Nimagen: $\kappa$ = 0.8837 ± 0.03753) still showing a high repeatability for Nimagen and Artic while Midnight v1 is not repeatable.

The results for nucleotide mutations were similar: the repeatability was very high for samples with qPCR Ct value < 25 (Artic v3: mean $\kappa$ = 0.9961 ± 0.002926, Midnight v1: mean $\kappa$ = 1 ± 0, Nimagen: mean $\kappa$ = 0.9742 ± 0.01683) but the kappa coefficient was lower for the sample with a Ct value >25 (Artic v3: $\kappa$ = 0.7581 ± 0.01951, Nimagen: $\kappa$ = 0.8287 ± 0.01903), especially for the Midnight v1 SOP which had a $\kappa$ coefficient = 0 ± 0.00003, indicating that it was not repeatable for samples with a qPCR Ct value > 25 (Fig. 1 right panel). This was due to the low read depth (<20) at most nucleotide positions for two repeats performed with the Midnight v1 SOP. As this depth was the minimum threshold set in the bio-informatics pipeline for nucleotide calling in the consensus sequence, and as the third repeat had a sufficient read depth at these positions, the analysis resulted in a complete mismatch. Although the repeatability is not significantly different between the three methods for samples with qPCR Ct value <25, the repeatability of the Midnight v1 SOP is significantly different when compared with both other methods for qPCR Ct value >25 (Supplementary table 3).

### Comparison of methods

The three SOPs described in this paper met the ISO15189 validation criterion for samples with qPCR Ct value < 25 which is the recommended exclusion criteria for analyses in clinical setting. Although the three methods were not powerful enough to analyze samples with a qPCR Ct value > 25 in clinical setting, sequencing results and strain typing can be obtained in non-clinical setting but should be interpreted with caution. Therefore, we compared the three methods using fifty-five samples without considering the Ct value exclusion criteria.

The samples were sequenced according to the three SOPs and compared at two levels: typing according to WHO guidelines and two databases (Pangolin and Nextclade), and SNVs identification.

For forty-five out of fifty-five samples with valid typing results including twenty-seven samples of the twenty-nine with a qPCR Ct value < 25, the WHO and NextClade nomenclatures show 100% concordance between the three methods. For Pangolin, which is a more stringent typing method, we observed five discrepancies between the SOPs. Among these, Pangolineage identified a variant directly originating from the closest ancestor identified for the other SOPs. We qualified these discrepancies as inaccuracies rather than real discrepancies. All samples presenting these inaccuracies had a qPCR Ct value >30 for the N-gene (Table 2).

**Table 2:** Comparison of typing results from three methods: Artic v3, Midnight v1 and Nimagen. Samples which could not be typed due to low coverage are marked as “no typing”. Typing results that do not match between the three methods are in bold.

Samples	N gene Ct Value	WHO			NextClade			Pangolin		
		Artic v3	Midnight v1	Nimagen	Artic v3	Midnight v1	Nimagen	Artic v3	Midnight v1	Nimagen
CV2031222739	12,4	Alpha	Alpha	Alpha	20I	20I	20I	B.1.1.7	B.1.1.7	B.1.1.7
CV8233434162	12,4	Delta	Delta	Delta	21J	21J	21J	AY.43	AY.43	AY.43
CV8144331679	12,7	Delta	Delta	Delta	21J	21J	21J	AY.43	AY.43	AY.43
CV8141142096	12,8	Delta	Delta	Delta	21J	21J	21J	AY.43	AY.43	AY.43
CV8144298741	12,9	Delta	Delta	Delta	21I	21I	21I	AY.9.2	AY.9.2	AY.9.2
CV8193173203	13,0	Delta	Delta	Delta	21J	21J	21J	AY.126	AY.126	AY.126
CV8144274792	15,3	Delta	Delta	Delta	21J	21J	21J	AY.4	AY.4	AY.4
CV2151486369	15,6	Gamma	Gamma	Gamma	20J	20J	20J	P.1.17.1	P.1.17.1	P.1.17.1
CV8144328245	15,9	Delta	Delta	Delta	21J	21J	21J	AY.121	AY.121	AY.121
CV8144272570	17,4	Delta	Delta	Delta	21J	21J	21J	AY.122	AY.122	AY.122
CV2239837205	17,8	Gamma	Gamma	Gamma	20J	20J	20J	P.1.16	P.1.16	P.1.16
CV2182385115	18,0	Alpha	Alpha	Alpha	20I	20I	20I	B.1.1.7	B.1.1.7	B.1.1.7
11617221	18,1	Delta	No Typing	Delta	21J	No Typing	21J	AY.4	No Typing	AY.4
CV8233401830	18,5	Delta	Delta	Delta	21J	21J	21J	AY.43	AY.43	AY.43
CV8144289849	18,6	Delta	Delta	Delta	21J	21J	21J	AY.43	AY.43	AY.43
CV8287216824	18,8	Delta	Delta	Delta	21J	21J	21J	AY.43	AY.43	AY.43
CV2151007635	19,2	Beta	Beta	Beta	20H	20H	20H	B.1.351	B.1.351	B.1.351
CV8144276008	19,3	Delta	Delta	Delta	21J	21J	21J	AY.122	AY.122	AY.122
CV2266749550	20,5	Beta	Beta	Beta	20H	20H	20H	B.1.351	B.1.351	B.1.351
CV8144298842	20,8	Delta	Delta	Delta	21J	21J	21J	B.1.617.2	B.1.617.2	B.1.617.2
CV8144268530	21,5	Alpha	Alpha	Alpha	20I	20I	20I	B.1.1.7	B.1.1.7	B.1.1.7
CV8144331881	21,5	Delta	Delta	Delta	21J	21J	21J	AY.43	AY.43	AY.43
11607860	21,8	Delta	No Typing	Delta	21J	No Typing	21J	AY.4.2	No Typing	AY.4.2
CV8144299650	22,0	Delta	Delta	Delta	21J	21J	21J	AY.98.1	AY.98.1	AY.98.1
CV2082164109	22,5	Gamma	Gamma	Gamma	20J	20J	20J	P.1.16	P.1.16	P.1.16
CV8144297832	22,5	Delta	Delta	Delta	21J	21J	21J	AY.43	AY.43	AY.43
CV8144281563	22,5	Delta	Delta	Delta	21J	21J	21J	AY.103	AY.103	AY.103
CV8144299751	22,8	Delta	Delta	Delta	21J	21J	21J	B.1.617.2	B.1.617.2	B.1.617.2
CV8144310461	23,1	Delta	Delta	Delta	21J	21J	21J	AY.126	AY.126	AY.126
11607450	25,9	Delta	No Typing	Delta	21J	No Typing	21J	AY.43	No Typing	AY.43
11616748	26,0	Delta	No Typing	Delta	21J	No Typing	21J	AY.122	No Typing	AY.122
CV2138930630	27,3	Gamma	Gamma	Gamma	20J	20J	20J	P.1.16	P.1.16	P.1.16
11607311	27,7	Delta	Delta	Delta	21J	21J	21J	AY.43	AY.43	AY.43
CV2266518871	28,0	Alpha	Alpha	Alpha	20I	20I	20I	B.1.1.7	B.1.1.7	B.1.1.7
11645231	28,6	Omicron	Omicron	Omicron	21K	21K	21K	BA.1.1.1	BA.1.1.1	BA.1.1.1
11617724	28,9	Delta	Delta	Delta	21J	21J	21J	AY.43	AY.43	AY.43
CV2031641758	29,0	Alpha	No Typing	Alpha	20I	No Typing	20I	B.1.1.7	No Typing	B.1.1.7
CV2150036827	29,2	Beta	Beta	Beta	20H	20H	20H	B.1.351	B.1.351	B.1.351
11606413	29,9	Delta	Delta	Delta	21J	21J	21J	AY.42	AY.42	AY.42
CV8144289445	30,3	Gamma	Gamma	Gamma	20J	20J	20J	**P.1**	**P.1**	**P.1.16**
11644679	31,2	Omicron	Omicron	Omicron	21K	21K	21K	BA.1.14	BA.1.14	BA.1.14
11607865	31,2	Delta	Delta	Delta	21J	21I	21J	**B.1.617.2**	**AY.58**	**AY.5.4**
11607538	31,3	Delta	Delta	Delta	21J	21J	21J	AY.46.6	AY.46.6	AY.46.6
11645100	32,1	Delta	Delta	Delta	21J	21J	21J	AY.43	AY.43	AY.43
11645096	32,4	Delta	Delta	Delta	21J	21J	21J	AY.43	AY.43	AY.43
11606205	32,5	Delta	Delta	Delta	21J	21J	21J	**AY.4**	**AY.4**	**AY.4.17**
11607928	32,6	Delta	No Typing	Delta	21J	No Typing	21J	B.1.617.2	No Typing	B.1.617.2
11598489	32,7	Delta	Delta	Delta	21J	21I	21I	B.1.617.2	B.1.617.2	B.1.617.2
11607727	32,8	Delta	Delta	Delta	21J	21J	21J	**B.1.617.2**	**AY.107**	**B.1.617.2**
11648633	34,3	No Typing	No Typing	Omicron	No Typing	No Typing	21K	No Typing	No Typing	BA.1
11606213	34,4	Delta	Delta	Delta	21J	21J	21J	**AY.4**	**AY.4.17**	**AY.4**
11645052	35,1	Delta	Delta	Delta	21J	21J	21J	AY.127	AY.127	AY.127
11606225	35,2	Delta	No Typing	No Typing	21J	No Typing	No Typing	AY.43	No Typing	No Typing
11647200	35,6	No Typing	No Typing	Omicron	No Typing	No Typing	21K	No Typing	No Typing	BA.1.14
11607445	36,0	No Typing	No Typing	No Typing	No Typing	No Typing	No Typing	No Typing	No Typing	No Typing

**Figure 2:**
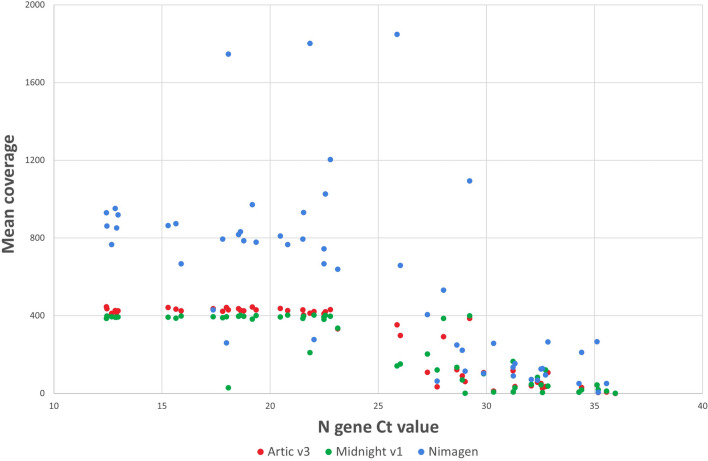
Evolution of the mean genome coverage according to the Ct value for the N-gene for the three methods: Artic v3 (red), Midnight v1 (green) and Nimagen (blue). Mean coverage is relatively steady for all samples with Ct value <25 and drops for samples with Ct value >25.

Ten samples sequenced with the Midnight v1 SOP could not be typed in any nomenclature due to insufficient coverage. Among these, three samples sequenced with the Artic SOP and two samples sequenced with the Nimagen SOP, respectively, could not be typed either. Eight of these ten samples had a N-gene qPCR Ct value >25 (Table 2), which led us to study the evolution of genome coverage as a function of viral load. We observed that for the three SOPs, the coverage was high for all samples with a N-gene qPCR Ct values <25 (96% and 90% of samples had a mean coverage above 380x for Artic v3 and Midnight v1 methods, respectively, and 90% of samples have a mean coverage above 600$\times$ for Nimagen method) but the sequencing coverage dropped drastically with the three SOPs for samples with a qPCR Ct value >25 (Fig. 2).

**Figure 3:**
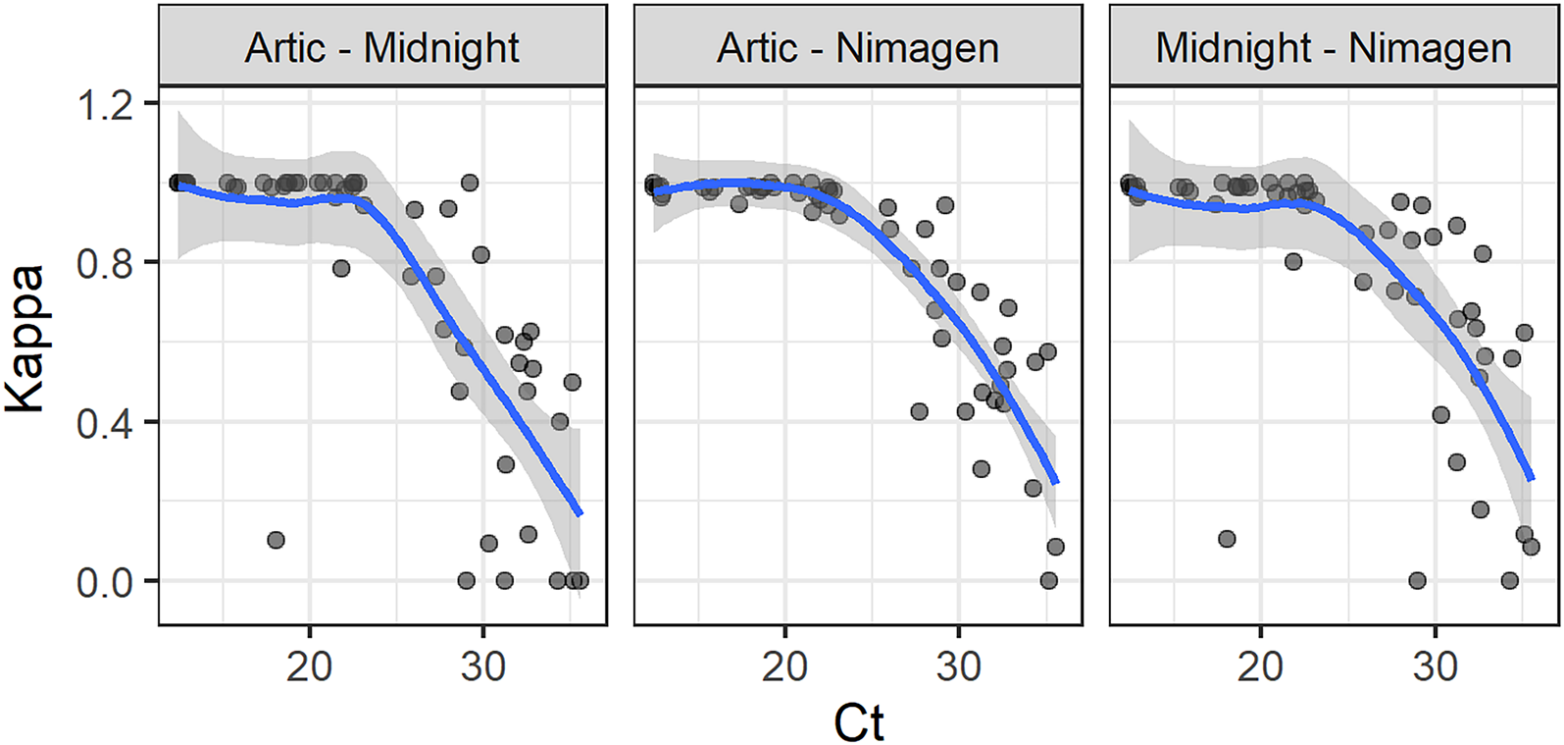
Evolution of Cohen’s kappa coefficient for the comparison of SNVs calls of three methods: Artic v3, Midnight v1 and Nimagen, compared two by two, depending on the Ct value for the N-gene of each sample. A kappa coefficient of 1 means a perfect match, while a kappa coefficient of 0 means a complete mismatch. The three methods call almost perfectly the same SNVs for samples with Ct value <25 (kappa coefficient close to 1) but SNVs calls differ more and more as the Ct value increases. The blue line is a smoothed curve based on the locally estimating scatterplot smoothing algorithm and the grey area represents the 95% confidence band.

We then compared the SNVs identified in each sample for one SOP vs the other using the Cohen’s kappa coefficient. Samples with a qPCR Ct value <25 had kappa coefficients close to or equal to 1, indicating a high concordance between both methods compared. For samples with a Ct value >25, the kappa coefficient decreased progressively with the increase of Ct value. This decrease was similar when comparing any method to one another (Fig. 3). However, all the mismatches observed can be explained by a too low read depth at the position of the mismatch for the method that did not identify the mutation (<20 reads for the Nanopore methods or <5 reads for the Illumina method). When, at that genomic position, the depth of reads was greater than 0 but below the previously mentioned cut-offs, we observed that the mutation was present in more than 70% of the reads, indicating that the discrepancies were not due to sequencing errors inherent to the methods but rather to the detection thresholds set in the bio-informatics pipelines.

### Time and cost

The three SOPs studied in this paper presented standard NGS library preparation procedures. In our hands, and to obtain optimal results, both ONT methods allowed to sequence forty-eight samples with a 9.4 flow cell and a GridION (Oxford Nanopore Technologies; Oxford, UK) in one run, while the Illumina method allowed to sequence twenty-four samples with an iSeq 100 i1 Reagent v2 cartridge (Illumina; CA, USA) in one run. The run, from reverse transcription to acquisition of fastq files, required 32h including 3h30 of hands-on-time with the Artic v3 SOP, 29h including 1h30 of hands-on-time for the Midnight v1 SOP and 29h30 including 2h30 of hands-on-time for the Nimagen SOP. It is worth noting that the sequencing time for both SOPs using Nanopore technology could be further reduced. The sequencing cost of one sample based on catalog prices, without considering extraction and plastic consumables, is the lowest for Midnight v1 SOP and the greatest for Nimagen SOP. Sequencing costs could be further reduced for the ONT procedures as that technology allows the re-use of sequencing flow cells. Any modifications or optimization of any of the procedures require at least a new verification of the method.

### Maintenance of bioinformatics pipelines

The bioinformatics pipelines were verified before their implementation in the analytical flow by carrying out three independent analyses of the raw data obtained after sequencing two samples. 100% typing concordance was obtained with the Nextclade and Pangolin databases. Following their implementation, each major update of one of the software involved in the pipelines resulted in a re-validation procedure that consisted of re-sequencing five samples for which data already analyzed using the latest version of the software were available. 100% typing concordance with the previous typing results (WHO, Nextclade and Pangolin) must be obtained. All updates and validation reports were logged into the quality management system of the lab.

## Discussion

The three methods evaluated in this study were validated for samples with a N-gene qPCR Ct value <25, fulfilling ISO15189 requirements. They generated reproducible, repeatable, and accurate data for whole genome sequencing of SARS-CoV-2 clinical samples. The ability to identify SARS-CoV-2 variants according to different nomenclatures was identical regardless of the method. Typing with the Pangolin database could result in minor discrepancies which were mainly due to the different versions of the database used rather than a technical error during sequencing procedures themselves. Another factor impacting the typing accuracy was the coverage. As already described in literature, our data confirmed that, in amplicon-based sequencing methods, the coverage was sensitive to viral load, the latter being correlated with the qPCR Ct value^36,37^. Samples with a qPCR Ct value >25 usually showed insufficient read depth which provided low reliability on some key mutations, which could lead to wrong or approximate lineage designation, highlighting the importance of sample selection criteria in the context of national surveillance programs using clinical samples^8,38^. Our data confirmed that the three methods validated here provided similar quality results and were adequate for epidemiological monitoring of SARS-CoV-2 using samples with N-gene qPCR CT value <25 in clinical setting.

For clonal relatedness determination in outbreak investigations requiring the exact detection of single nucleotide variants (SNVs), our results suggested that all three methods could be used when analyzing samples with high viral load (qPCR Ct value <25). However, our data indicated that for samples with low viral load (qPCR Ct value >25), a method using smaller amplicons (Artic v3 and Nimagen) should be preferred as they exhibit better reproducibility and repeatability, and a higher mean coverage of the genome. This variability in coverage for samples with a low viral load had already been reported by Freed et al. Nevertheless, they have shown that it was possible to reach good quality results by generating more sequencing data than for samples with high or medium viral loads. This was not achieved in our study as we sequenced all samples under the same conditions. It has also been shown that in order to effectively detect SNVs and by extension reach a minimum coverage of 400x with an amplicon-based NGS technique, the material used for reverse transcription should contain at least 1,000 copies of viral RNA^39,40^.

All three methods described in this paper are amplicon-based methods. They efficiently enabled the enrichment of target sequences to obtain optimal results for a wide range of samples. However, they were sensitive to substitutions, deletions, insertions, in the sequence to which the primers hybridized. Since RNA viruses, including SARS-CoV-2, regularly present this type of event in their genome^10^, it is essential to identify dropouts of certain amplicons and to regularly adapt the primers used in the SOPs according to the evolution of the virus. The Midnight v1 amplification method used a lower number of primers making it less sensitive to dropouts. Nonetheless, if dropouts still occur, the region that will no longer be covered will be much larger with the Midnight v1 SOP than with the Artic or Nimagen SOPs. However, the primer design of the three SOPs allows for the amplification of overlapping fragments (Supplementary Figure 1) enabling rapid identification of a mutation in the complementary sequence of a primer using open access tools such as Integrative Genomics Viewer^41^.

In several countries, including Belgium, strain-specific PCRs (or VOC PCR) were temporarily implemented for rapid and inexpensive monitoring of VOCs such as during the emergence of the alpha and omicron BA.1 variants, which were characterized by an S-gene target failure detectable by PCR kits^42^. These PCR mutation markers quickly reached their limits as they suffered from the high mutation rate of the virus, thus requiring too frequent update of PCR primers, leaving NGS as the gold standard technique for the detection and monitoring of VOCs in surveillance programs. In that context, and despite its inferior sequencing quality for low viral load samples, the Midnight v1 method should not be neglected. Its technical protocol is very simple and quick to set up compared to both other methods presented here, which are much time consuming and tedious for technical staff. It is also less expensive and therefore may be the ideal method for large-scale monitoring, especially in low-income countries.

In 2028, all European diagnostic labs will have to comply with the new European regulation for medical devices and *in vitro* diagnostics that came into effect in 2022 and aims to standardize further clinical analyses and increase patient safety^17,18^. A major requirement of the IVDR is that all laboratory developed test (e.g. NGS typing methods for emerging and rapidly spreading pathogens) must be accredited by a national accreditation body, entailing a full validation of the method fulfilling the ISO15189 standards^19^. This study is a first step toward the implementation of the IVDR. Nevertheless, this race to standardization and safety might reduce the ability for medical laboratories to provide cutting edge diagnostics. In molecular microbiology, typing of rapidly evolving viruses during an epidemic or pandemic requires flexibility such as the adaptation of primer sets for amplicon-based methods or the regular updates of typing databases. In molecular pathology, the constant evolution of the medical practice and the expanding knowledge about tumor development and acquisition of resistance to treatments require regular update of DNA mutation and RNA fusion panels for patients to benefit rapidly from the latest knowledge in the field. Any major change to a validated procedure (e.g.: reagent composition, software version, database) implying a new complete validation, compliance with the IVDR will be very challenging, time consuming, and costly for medical laboratories willing to stay at the forefront of their practice.

In conclusion, the three methods presented in this paper met, in our hands, all the requirements of the ISO15189 standard for the establishment of an epidemiological typing method in clinical setting and can be used with equal confidence in the framework of SARS-CoV-2 surveillance programs. However, methods using shorter amplicons should be preferred when working with samples with low viral load. To the best of our knowledge, this study is the first to propose a complete validation process for SARS-CoV-2 Whole Genome Sequencing SOPs complying with ISO15189 standards and leading to the accreditation of the methods by a national accreditation body, a first step towards IVDR implementation in clinical setting.

## Supplementary Information

Supplementary Information.

## Data Availability

The datasets generated during the current study are available in the BioSample repository, accession PRJNA954542. https://www.ncbi.nlm.nih.gov/bioproject/954542.

## References

[1] Weiss SR, Leibowitz JL (2011). Coronavirus pathogenesis. Adv. Virus Res..

[2] Cui J, Li F, Shi Z-L (2019). Origin and evolution of pathogenic coronaviruses. Nat. Rev. Microbiol..

[3] González-Candelas F (2021). One year into the pandemic: Short-term evolution of SARS-CoV-2 and emergence of new lineages. Infect. Genet. Evol..

[4] Burki T (2021). Understanding variants of SARS-CoV-2. The Lancet.

[5] Otto SP (2021). The origins and potential future of SARS-CoV-2 variants of concern in the evolving COVID-19 pandemic. Curr. Biol..

[6] Chen Y (2022). Emerging SARS-CoV-2 variants: Why, how, and what’s next?. Cell Insight.

[7] (COG-UK), C. An integrated national scale SARS-CoV-2 genomic surveillance network. *The Lancet Microbe* **1**, e99–e100. 10.1016/S2666-5247(20)30054-9 (2020). 10.1016/S2666-5247(20)30054-9 PMC7266609 32835336

[8] Cuypers L (2022). Two years of genomic surveillance in Belgium during the SARS-CoV-2 pandemic to attain country-wide coverage and monitor the introduction and spread of emerging variants. Viruses.

[9] Zhu N (2020). A novel coronavirus from patients with pneumonia in China, 2019. N. Engl. J. Med..

[10] Zhou P (2020). A pneumonia outbreak associated with a new coronavirus of probable bat origin. Nature.

[11] Lu R (2020). Genomic characterisation and epidemiology of 2019 novel coronavirus: Implications for virus origins and receptor binding. The Lancet.

[12] Rambaut A (2020). Preliminary genomic characterisation of an emergent SARS-CoV-2 lineage in the UK defined by a novel set of spike mutations.

[13] Harvey WT (2021). SARS-CoV-2 variants, spike mutations and immune escape. Nat. Rev. Microbiol..

[14] Berkhout B, Herrera-Carrillo E (2022). SARS-CoV-2 Evolution: On the Sudden Appearance of the Omicron Variant. J. Virol..

[15] Gargis AS, Kalman L, Lubin IM (2016). Assuring the quality of next-generation sequencing in clinical microbiology and public health laboratories. J. Clin. Microbiol..

[16] Thelen MH, Huisman W (2018). Harmonization of accreditation to ISO15189. Clin. Chem. Lab. Med. (CCLM).

[17] Parliament, E. & Council. REGULATION (EU) 2017/745 on medical devices, amending directive 2001/83/EC, regulation (EC) no 178/2002 and regulation (EC) no 1223/2009 and repealing council directives 90/385/EEC and 93/42/EEC. Tech. Rep., European Parliament and Council (2017).

[18] Parliament, E. & Council. REGULATION (EU) 2017/746 on in vitro diagnostic medical devices and repealing directive 98/79/EC and commission decision 2010/227/EU. Tech. Rep., European Parliament and Council (2017).

[19] Vanstapel FJ (2023). ISO 15189 is a sufficient instrument to guarantee high-quality manufacture of laboratory developed tests for in-house-use conform requirements of the European In-Vitro -Diagnostics Regulation: Joint opinion of task force on European regulatory affairs and working group accreditation and ISO/CEN standards of the European Federation of Clinical Chemistry and Laboratory Medicine. Clin. Chem. Lab. Med. (CCLM).

[20] Jacot D, Pillonel T, Greub G, Bertelli C (2021). Assessment of SARS-CoV-2 genome sequencing: Quality criteria and low-frequency variants. J. Clin. Microbiol..

[21] Wegner F (2022). External quality assessment of SARS-CoV-2 sequencing: An ESGMD-SSM pilot trial across 15 European laboratories. J. Clin. Microbiol..

[22] Tyson, J. R. *et al.* Improvements to the ARTIC multiplex PCR method for SARS-CoV-2 genome sequencing using nanopore. preprint, *Genomics* (2020). 10.1101/2020.09.04.283077.

[23] Freed, N. E., Vlková, M., Faisal, M. B. & Silander, O. K. Rapid and inexpensive whole-genome sequencing of SARS-CoV-2 using 1200 bp tiled amplicons and Oxford Nanopore Rapid Barcoding. *Biol. Methods Protoc.* **5**, bpaa014. 10.1093/biomethods/bpaa014 (2020). 10.1093/biomethods/bpaa014 PMC7454405 33029559

[24] Coolen JP (2021). SARS-CoV-2 whole-genome sequencing using reverse complement PCR: For easy, fast and accurate outbreak and variant analysis. J. Clin. Virol..

[25] Cotten, M., Lule Bugembe, D., Kaleebu, P. & Phan, M. V.T. Alternate primers for whole-genome SARS-CoV-2 sequencing. *Virus Evol.* **7**, veab006. 10.1093/ve/veab006 (2021). 10.1093/ve/veab006 PMC7928614 33841912

[26] Itokawa K, Sekizuka T, Hashino M, Tanaka R, Kuroda M (2020). Disentangling primer interactions improves SARS-CoV-2 genome sequencing by multiplex tiling PCR. PLoS ONE.

[27] Kieser RE (2020). Reverse complement PCR: A novel one-step PCR system for typing highly degraded DNA for human identification. Forens. Sci. Int. Genet..

[28] Loman, N., Rowe, W. & Rambaut, A. ARTIC-nCoV-bioinformaticsSOP-v1.1.0.

[29] Lee JY (2021). Comparative evaluation of Nanopore polishing tools for microbial genome assembly and polishing strategies for downstream analysis. Sci. Rep..

[30] Aksamentov, I., Roemer, C., Hodcroft, E. & Neher, R. Nextclade: Clade assignment, mutation calling and quality control for viral genomes. *J. Open Source Softw.* **6**, 3773. 10.21105/joss.03773 (2021).

[31] O’Toole, A. *et al.* Assignment of Epidemiological Lineages in an Emerging Pandemic Using the Pangolin Tool. *Virus Evol.* veab064, 10.1093/ve/veab064 (2021). 10.1093/ve/veab064 PMC8344591 34527285

[32] ISO 15189:2012 Medical laboratories - Requirements for quality and competence. Tech. Rep., International Organization for Standardization (2012).

[33] Grangeia HB, Silva C, Simões SP, Reis MS (2020). Quality by design in pharmaceutical manufacturing: A systematic review of current status, challenges and future perspectives. Eur. J. Pharm. Biopharm..

[34] Nunnally BK, Turula VE, Sitrin RD (2015). Vaccine Analysis: Strategies, Principles, and Control.

[35] Coleman, W. & Tsongalis, G. Chapter 2 - laboratory approaches in molecular pathology-the polymerase chain reaction. In *Laboratory Approaches in Molecular Pathology-The Polymerase Chain Reaction. In: Diagnostic Molecular Pathology.*, 15–23 (Academic Press, Cambridge, 2017).

[36] Charre, C. *et al.* Evaluation of NGS-based approaches for SARS-CoV-2 whole genome characterisation. *Virus Evolution* **6**, veaa075. 10.1093/ve/veaa075 (2020). 10.1093/ve/veaa075 PMC7665770 33318859

[37] Tshiabuila D (2022). Comparison of SARS-CoV-2 sequencing using the ONT GridION and the Illumina MiSeq. BMC Genom..

[38] Risk assessment group, R. Recommandations pour la sélection d’échantillons en vue du séquençage du génome complet. Tech. Rep., Risk assessment group, RAG (2021).

[39] Grubaugh ND (2019). An amplicon-based sequencing framework for accurately measuring intrahost virus diversity using PrimalSeq and iVar. Genome Biol..

[40] Kubik S (2021). Recommendations for accurate genotyping of SARS-CoV-2 using amplicon-based sequencing of clinical samples. Clin. Microbiol. Infect..

[41] Robinson JT (2011). Integrative genomics viewer. Nat. Biotechnol..

[42] Cuypers L (2022). Nationwide harmonization effort for semi-quantitative reporting of SARS-CoV-2 PCR test results in Belgium. Viruses.

