# Supplementary material for: Minimal requirements for ISO15189 validation and accreditation of three next generation sequencing procedures for SARS-CoV-2 surveillance in clinical setting
Source: Sci Rep. 2023 Apr 28;13:6934. doi: 10.1038/s41598-023-34088-w (PMC10140720; doi:10.1038/s41598-023-34088-w)
Supplement: Supplementary file 1 — Supplementary Information. [file 41598_2023_34088_MOESM1_ESM.pdf]

Supplementary informations

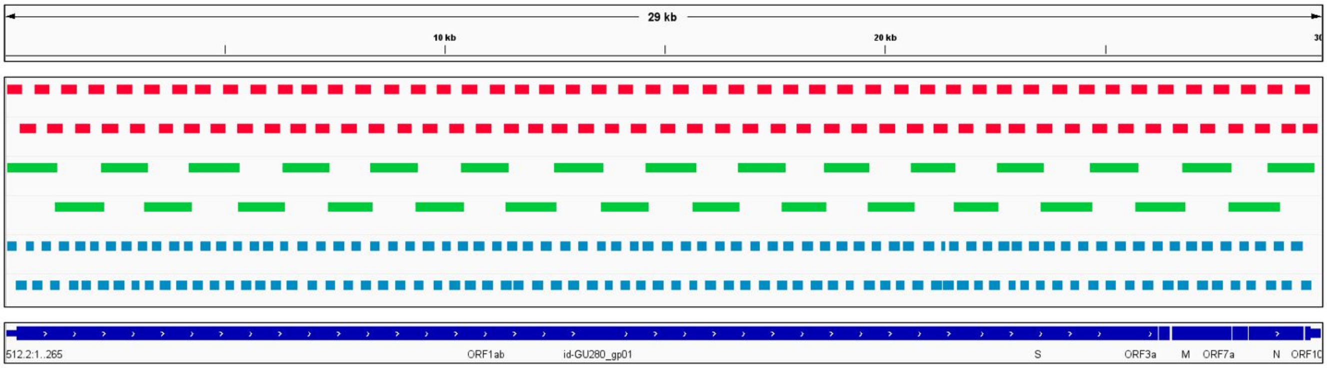

Supplementary Figure 1. Representation of the amplicons generated by the three methods: Artic v3 (red), Midnight v1 (green) and Nimagen (blue) (middle panel) alongside their complementary sequences on the SARS-CoV-2 genome NC_045512.2 (top panel) and the RefSeq gene (bottom panel) shown in Integrative Genome Viewer (IGV).

Supplementary Table 1. The 5M table evaluates uncertainties of measures by identifying the risk factors and the means put in place to limit their effects on the quality of the results

5M	Critical points	Points to control	Means of control
**Management/ Medium**	Laboratory	Management of molecular biology rooms	- Rooms have dedicated functions: pre-PCR, extraction or post-PCR<br>- Pre-PCR room is dedicated to PCR mixes making and reagents storage. No primary samples or nucleic acid can enter this room<br>- Extraction room is dedicated to nucleic acid extraction from primary samples and adding of nucleic acids in PCR mixes<br>- Post-PCR room is dedicated to PCR and library preparation<br>- After each manipulation, work tables are cleaned with DNA-Erase™ Decontamination Solution (MP Biomedicals; Irvine, CA)
	Reagents	Reagents conservation conditions	Monitoring storage devices temperature
	Devices	Devices environment	Monitored room temperature
**Machine**	Devices malfunction	Maintenances	- Daily and yearly maintenance for the Hamilton Microlab STARlet automated liquid handling system (Accuramed; Halen, Belgium) and the KingFisher Flex automated system (Thermo Fisher Scientific; MA, USA). There are back-up devices.<br>- Quarterly and yearly maintenance for thermocyclers<br>- Yearly maintenance for iSeq100 (Illumina; San Diego, CA, USA) and GridIon (Oxford Nanopore Technologies; Oxford, UK)
	Softwares	Version tracking	Verification of the method after each major software update.<br>Tracking sheet for softwares versions.
**Material**	Reagents validity	Batch verification before use	Technical sheet checking, reagent checking according to technical sheet and sector manager approval
	Technical sheet	Notice check	Checking of technical sheet and tracking in quality management system
**Method**	Relevance and disponibility of operating procedures (OP)	Document management	Reviewing of SOP every two years. Each review is tracked in the quality management software. Each SOP is available in the quality management system.
	Results	Acceptance criteria of whole sequence	- Quality score of sequencing<br>- Coverage
		Interpretation criteria	Interpretation by independent databases:<br>- WHO<br>- NextClade<br>- Pangolin
		Results encoding	Manual encoding in electronic patient record
	Procedure	Diffusion	Results protocols are issued daily after validation by the biologists
**Manpower**	Technicians	Training	- Trained and authorized staff<br>- Disseminated, known and applied procedure

Supplementary Table 2. Reproducibility of typing results for the three methods: Artic v3, Midnight v1 and Nimagen. The reproducibility is represented in percentage of consistency between three independent sequencing runs for each sample.

Methods	Classification	Samples	CV8233434162	CV8144272570	CV8287216824	CV2266749550	CV2082164109	CV8144297832	CV2031641758
		**N gene Ct value**	12,5	17,4	18,8	20,5	22,5	22,5	29,0
**Artic v3**	**WHO**	Run 1	Delta	Delta	Delta	Beta	Gamma	Delta	Alpha
		Run 2	Delta	Delta	Delta	Beta	Gamma	Delta	Alpha
		Run 3	Delta	Delta	Delta	Beta	Gamma	Delta	Alpha
		Reproducibility (%)	100	100	100	100	100	100	100
	**NextClade**	Run 1	21J	21J	21J	20H	20J	21J	20I
		Run 2	21J	21J	21J	20H	20J	21J	20I
		Run 3	21J	21J	21J	20H	20J	21J	20I
		Reproducibility (%)	100	100	100	100	100	100	100
	**Pangolin**	Run 1	AY.43	AY.122	AY.43	B.1.351	P.1.16	AY.43	B.1.1.7
		Run 2	AY.43	AY.122	AY.43	B.1.351	P.1.16	AY.43	B.1.1.7
		Run 3	AY.43	AY.122	AY.43	B.1.351	P.1.16	AY.43	B.1.1.7
		Reproducibility (%)	100	100	100	100	100	100	100
**Midnight v1**	**WHO**	Run 1	Delta	Delta	Delta	Beta	Gamma	Delta	No typing
		Run 2	Delta	Delta	Delta	Beta	Gamma	Delta	No typing
		Run 3	Delta	Delta	Delta	Beta	Gamma	Delta	No typing
		Reproducibility (%)	100	100	100	100	100	100	-
	**NextClade**	Run 1	21J	21J	21J	20H	20J	21J	No typing
		Run 2	21J	21J	21J	20H	20J	21J	No typing
		Run 3	21J	21J	21J	20H	20J	21J	No typing
		Reproducibility (%)	100	100	100	100	100	100	-
	**Pangolin**	Run 1	AY.43	AY.122	AY.43	B.1.351	P.1.16	AY.43	No typing
		Run 2	AY.43	AY.122	AY.43	B.1.351	P.1.16	AY.43	No typing
		Run 3	AY.43	AY.122	AY.43	B.1.351	P.1.16	AY.43	No typing
		Reproducibility (%)	100	100	100	100	100	100	-
**Nimagen**	**WHO**	Run 1	Delta	Delta	Delta	Beta	Gamma	Delta	Alpha
		Run 2	Delta	Delta	Delta	Beta	Gamma	Delta	Alpha
		Run 3	Delta	Delta	Delta	Beta	Gamma	Delta	Alpha
		Reproducibility (%)	100	100	100	100	100	100	100
	**NextClade**	Run 1	21J	21J	21J	20H	20J	21J	20I
		Run 2	21J	21J	21J	20H	20J	21J	20I
		Run 3	21J	21J	21J	20H	20J	21J	20I
		Reproducibility (%)	100	100	100	100	100	100	100
	**Pangolin**	Run 1	AY.43	AY.122	AY.43	B.1.351	P.1.16	AY.43	B.1.1.7
		Run 2	AY.43	AY.122	AY.43	B.1.351	P.1.16	AY.43	B.1.1.7
		Run 3	AY.43	AY.122	AY.43	B.1.351	P.1.16	AY.43	B.1.1.7
		Reproducibility (%)	100	100	100	100	100	100	100

Supplementary Table 3. Estimate (Mean of kappa index or difference between two kappa indexes), SE (standard error of the estimate) and P-value for inter-run reproducibility and intra-run repeatability for the three methods: Artic, Midnight and Nimagen for different Ct ranges and for comparison between reproducibility and repeatability of these methods in pairs. Estimates and standard errors are computed from a linear mixed model with Kappa as the dependent variable, type of analysis (intra- or inter-run), Ct value and methods as the independent fixed variables and a random intercept for each sample ID. The P-values are adjusted with the Bonferroni method.

**Type**	**Ct range**		**Artic**	**Midnight**	**Nimagen**	**Artic - Midnight**	**Artic - Nimagen**	**Midnight - Nimagen**
**Inter-run**	(10,20]	Estimate	0,99	1,00	0,93	-0,01	0,07	0,07
		SE	0,02	0,02	0,02	0,02	0,02	0,02
		P-value				1,0	0,0033	0,00061
	(20,25]	Estimate	1,00	1,01	0,97	-0,01	0,03	0,04
		SE	0,02	0,02	0,02	0,02	0,02	0,02
		P-value				1,0	1,0	0,44
	(25,40]	Estimate	0,69	0,00	0,77	0,69	-0,08	-0,77
		SE	0,03	0,03	0,03	0,03	0,03	0,03
		P-value				**<0,0001**	0,20	**<0,0001**
**Intra-run**	(10,20]	Estimate	0,97	0,98	0,94	-0,01	0,03	0,04
		SE	0,02	0,02	0,02	0,02	0,02	0,02
		P-value				1,0	1,0	0,93
	(20,25]	Estimate	1,01	1,01	0,99	0,00	0,02	0,02
		SE	0,03	0,03	0,02	0,03	0,03	0,03
		P-value				1,0	1,0	1,0
	(25,40]	Estimate	0,76	0,00	0,83	0,76	-0,07	-0,83
		SE	0,03	0,03	0,03	0,03	0,03	0,03
		P-value				**<0,0001**	0,34	**<0,0001**

Supplementary Table 4. Repeatability of typing results for the three methods: Artic v3, Midnight v1 and Nimagen. The repeatability is represented in percentage of consistency between three repeats in one sequencing run.

Methods	*Classification*	Samples	*CV8233434162*	*CV8144272570*	*CV8287216824*	*CV2266749550*	*CV2082164109*	*CV8144297832*	*CV2031641758*	*CV8144331881*	*CV8144299650*	*CV8144281563*
		**N gene Ct value**	12,5	17,4	18,8	20,5	22,5	22,5	29,0	21,5	22,0	22,5
**Artic v3**	**WHO**	Repeat 1	Delta	Delta	Delta	Beta	Gamma	Delta	Alpha	-	-	-
		Repeat 2	Delta	Delta	Delta	Beta	Gamma	Delta	Alpha	-	-	-
		Repeat 3	Delta	Delta	Delta	Beta	Gamma	Delta	Alpha	-	-	-
		Repeatability (%)	100	100	100	100	100	100	100	-	-	-
	**NextClade**	Repeat 1	21J	21J	21J	20H	20J	21J	20I	-	-	-
		Repeat 2	21J	21J	21J	20H	20J	21J	20I	-	-	-
		Repeat 3	21J	21J	21J	20H	20J	21J	20I	-	-	-
		Repeatability (%)	100	100	100	100	100	100	100	-	-	-
	**Pangolin**	Repeat 1	AY.43	AY.122	AY.43	B.1.351	P.1.16	AY.43	B.1.1.7	-	-	-
		Repeat 2	AY.43	AY.122	AY.43	B.1.351	P.1.16	AY.43	B.1.1.7	-	-	-
		Repeat 3	AY.43	AY.122	AY.43	B.1.351	P.1.16	AY.43	B.1.1.7	-	-	-
		Repeatability (%)	100	100	100	100	100	100	100	-	-	-
**Midnight v1**	**WHO**	Repeat 1	Delta	Delta	Delta	Beta	Gamma	Delta	No typing	-	-	-
		Repeat 2	Delta	Delta	Delta	Beta	Gamma	Delta	No typing	-	-	-
		Repeat 3	Delta	Delta	Delta	Beta	Gamma	Delta	No typing	-	-	-
		Repeatability (%)	100	100	100	100	100	100	-	-	-	-
	**NextClade**	Repeat 1	21J	21J	21J	20H	20J	21J	No typing	-	-	-
		Repeat 2	21J	21J	21J	20H	20J	21J	No typing	-	-	-
		Repeat 3	21J	21J	21J	20H	20J	21J	No typing	-	-	-
		Repeatability (%)	100	100	100	100	100	100	-	-	-	-
	**Pangolin**	Repeat 1	AY.43	AY.122	AY.43	B.1.351	P.1.16	AY.43	No typing	-	-	-
		Repeat 2	AY.43	AY.122	AY.43	B.1.351	P.1.16	AY.43	No typing	-	-	-
		Repeat 3	AY.43	AY.122	AY.43	B.1.351	P.1.16	AY.43	No typing	-	-	-
		Repeatability (%)	100	100	100	100	100	100	-	-	-	-
**Nimagen**	**WHO**	Repeat 1	Delta	-	Delta	-	Gamma	-	Alpha	Delta	Delta	Delta
		Repeat 2	Delta	-	Delta	-	Gamma	-	Alpha	Delta	Delta	Delta
		Repeat 3	Delta	-	Delta	-	Gamma	-	Alpha	Delta	Delta	Delta
		Repeatability (%)	100	-	100	-	100	-	100	100	100	100
	**NextClade**	Repeat 1	21J	-	21J	-	20J	-	20I	21J	21J	21J
		Repeat 2	21J	-	21J	-	20J	-	20I	21J	21J	21J
		Repeat 3	21J	-	21J	-	20J	-	20I	21J	21J	21J
		Repeatability (%)	100	-	100	-	100	-	100	100	100	100
	**Pangolin**	Repeat 1	AY.43	-	AY.43	-	P.1.16	-	B.1.1.7	AY.43	AY.98.1	AY.103
		Repeat 2	AY.43	-	AY.43	-	P.1.16	-	B.1.1.7	AY.43	AY.98.1	AY.103
		Repeat 3	AY.43	-	AY.43	-	P.1.16	-	B.1.1.7	AY.43	AY.98.1	AY.103
		Repeatability (%)	100	-	100	-	100	-	100	100	100	100
